# Progress in Medicine

**Published:** 1897-06-19

**Authors:** 


### Progress in Medicine.

### DISEASES OF THE LUNGS.—TUBERCULOSIS.

*(Continued from page 184.)*

**Anti-tuberculous Serum.**—The use of the anti-toxic serum for tuberculous diseases practically dates from its introduction by C. Maragliano. Last year he published[5] his method of preparing the serum, and recorded some researches upon the anti-toxin it contains. In order to obtain it animals are inoculated with toxic substances derived from virulent cultures of human tuberculosis. These toxic materials are prepared in two distinct groups: First, a liquid, A, is procured by evaporating the culture at 100 deg. on a water-bath and filtering it through a Chamberland filter, as is done in the preparation of tuberculin. Second, a liquid, B, is obtained by passing the unwarmed culture through the Chamberland filter and concentrating it in a vacuum of 30 deg. Liquid A contains the bacterial proteins, and liquid B the bacterial toxins. Animals which can furnish serum (horses) are inoculated with a mixture of three parts of the liquid A and one part of the liquid B. At the outset two milligrammes of the mixture are injected for each kilogramme of weight of the animal, this dose being increased one milligramme a day and per kilogramme until forty to fifty milligrammes for each kilogramme are given. After this the same quantity is inoculated for six months, and at the end of this time the animal is immunised, for it then resists the inoculation of considerable quantities of toxic materials and the injection of cultures virulent enough to kill the control animal. Three or four weeks after the cessation of the injections there will be considerable toxic material in the blood, and the animal is then bled, and thus the anti-tuberculous serum is obtained. This serum contains specific anti-toxins which neutralise the action of the tuberculous poisons. Experiments upon healthy guinea-pigs and upon tuberculous guinea-pigs demonstrate the fact. This serum possesses a bactericidal property in the test-tube in company with the bacillus tuberculosis.

**The New Tuberculin.**—We must now refer to the new tuberculin of Koch,[6] which he has recently introduced after extended clinical and laboratory investigations. The old tuberculin was a glycerin extract of a tubercle culture. This he originally claimed as a valuable diagnostic agent, and he still claims that it is a most effectual means of diagnosis at a very early period, and of value before any of the characteristic physical signs have appeared, both in man and animals.

Now the new tuberculin is prepared by a mechanical destruction of the bacilli. By triturating dried cultures in an agate mortar, adding distilled water, and subsequently using a powerful centrifugal machine, he eliminates the "sebacic acids." A whitish, opalescent fluid is thus obtained, the sediment containing the *débris* of the bacilli and any bacilli not destroyed by the trituration. The sediment is dried and again submitted to the same process, the second sediment being similarly dealt with, and so on till only a series of clear opalescent fluids remain. The first fluid obtained he calls tuberculin O, and this, treated with glycerin, contains the substances soluble in glycerin, being, therefore, similar to the old tuberculin, and, like the old tuberculin, causes reactions, but does not confer immunity. The second fluid he calls tuberculin R, and glycerin, on being added to this, produces a cloudiness due to the presence of substances not soluble in glycerin. This second fluid, or tuberculin R, is the new tuberculin.

Koch's opening remarks on the question of immunity are interesting and important. He points out that persons may have tuberculosis for years without acquiring immunity, and that even those who have been cured are liable to re-infection. Now, he believes that in the chronic form this is owing to the lack of opportunity for the absorption of the bacilli or their products. The bacilli are present in the tissue in small numbers, and are generally surrounded by dead tissues, or if numerous, as in cavities, the conditions are too unfavourable for the production of immunity by slow absorption. On the other hand, in acute miliary tuberculosis, he believes that the bacilli are at first present in large numbers, and after a short interval these largely disappear, which Koch attributes to an acquired immunity, coming, however, too late to save the patient. If, he argues, we can in any way imitate what occurs in acute tuberculosis, and at the same time avoid the fatality of the acute affection, we shall confer immunity upon the organism, and this problem Koch believes he has solved by his new tuberculin. The remedy is given just in the same way as the old tuberculin, beginning with a very small dose, which is increased gradually till the maximum dose is attained. The fluid is said to contain ·008 grms. of the active principle to each cubic centimetre. It is diluted with salt solution when used, the initial dose being one five-hundredth of a milligram. If, however, a reaction follows, the dose should be still further reduced. The injections are given every alternate day, the dose being gradually increased during a period of two or three weeks to 20 milligrammes. If the temperature is elevated another injection must not be given till it has subsided.

**Clinical Results of the New Methods.**—It will be noted that while the tuberculins aim at teaching the organism to produce its own anti-toxin, and thus acquire a more or less persistent immunity, the anti-toxic serums have for their object the development of the anti-toxin in the body of an insusceptible animal, and that ready-made it is injected into the tissues of the infected patient. Sufficient data to compare the relative value of these divergent methods are not yet to hand; indeed, any claims advanced for either method must be received with some reserve, owing to the nature of the disease itself.

Frances Carlucci,[7] of New York, reports five cases treated with Maragliano serum. He remarks that, whatever may be its action, there is the essential fact that the serum neutralises the action of the tuberculin, for it is well known that an injection of three milligrammes of tuberculin causes a reaction in a patient suffering from tuberculosis with no fever. Now if the same patient be given first an injection of one c.c. of serum and one of three milligrammes of tuberculin afterwards, the reaction is stopped. Evidently this fact, says he, shows that the Maragliano serum contains defensive substances. Briefly—Case I.: A woman, aged 44, with involvement of both apices, elevated temperature, copious expectoration crowded with bacilli of tubercle, after 32 injections showed a marvellous improvement in regard to cough, appetite, and general condition. The râles had disappeared from the left apex, but the right, where there was a cavity, was unchanged. "This amelioration brought about by the serum could not be obtained by a previous treatment with creosote during five months." Case II.: Man, aged 25, with recent pleurisy, temperature, expectoration crowded with bacilli, after 50 c.c. of serum had greatly improved. The cough was diminished, expectoration less abundant, and showing less bacilli. Night sweats were diminished also. Case III.: Aged 24, in similar general condition, with extensive involvement of the right upper lobe, bacilli in sputum, &c. After 44 injections of 1 c.c. each, showed even greater improvement, and the râles disappeared from the right apex. Cases IV. and V. were equally satisfactory.

Carlucci concludes that (*a*) the serum is innocuous; (*b*) the serum causes the drying up of broncho-pneumonic foci; (*c*) it improves the appetite and increases the weight, so that the general return of physical force is felt; (*d*) the effect of the serum is gradual. He adds that in Italy more than three hundred cases have been treated with the serum with very satisfactory results. The same results have been obtained by Gerhardt in Berlin, and by Babes in Paris.

Again, Renzi[8] reports a number of cases. In three patients, whose condition was rather serious, 10 c.cm. were injected at one time, and repeated at intervals of three to eight days for twice or three times. In all of these when so treated by the larger dosage the injection was well borne, and no local bad effect followed with the exception of slight tumefaction on one occasion. In all of these there was increase of appetite and a certain sense of well-being, so that it was possible to increase the daily amount of food. In 22 instances the weight increased in 12, remained stationary in one, diminished in nine. Inspiratory force increased in 14, remained stationary in eight. The temperature increased in 10, remained stationary in two, diminished in 10. The amount of sputum and the quantity of bacilli diminished in 10 and 13 respectively, being stationary in the rest. In all instances the moist sounds gradually diminished, disappearing in two. His experience also is that no inconvenient results follow its use, and even the appearance of a very slight amount of serum albumen in the urine does not modify this opinion. At the same time he is careful to point out that no good may be obtained by employing too large a dose. Regnier[9] says the serum has no special pyretogenous action. All depends on the dosage employed. In the usual therapeutic doses to which the patient is accustomed there is no elevation of temperature, nor is there any painful or local reaction. Doses above 2 c.cm. may cause transitory elevation of temperature, which may at times reach a considerable figure, more especially in the case of tuberculosis subjects. This may last two or three days, and is not accompanied by any local manifestation in the affected pulmonary areas. The different action on the temperature does not seem to depend on the severity of the disease, but rather upon individual peculiarities which may be looked upon as independent of the tuberculous lesions, but depending upon other factors, amongst which the influence of the nervous system is preponderant. These injections cause augmentation in the number of white blood corpuscles, which may be considerable. The red corpuscles and hæmoglobin increase with the general improvement. The urine shows no appreciable modification except in some cases where large doses have been employed, when there may be peptonuria but never glycosuria nor albumen, and no increase in the toxicity. The influence on nutrition is, as a rule, satisfactory. After a few injections, appetite increases, as does also the weight of the body proportionately. In cases of tubercle with elevation of temperature, small doses—1 to 2 c.cm.—do not produce any immediate alterations in the temperature; but after a few days the pyrexia decreases, especially when it was well marked Larger quantities—5 to 10 c.cm.—raise the temperature for a couple of days. Afterwards the elevation produced diminishes, and the patients become apyretic. The injections produce an evident effect upon the areas of tuberculous broncho-pneumonia. In general, at the end of a month, sometimes sooner, these clear up, no more râles are heard, and breath sounds remain slightly harsh. These satisfactory results are obtained when there are only small numbers of associated micro-organisms. Cough disappears relatively to these changes, expectoration diminishes or disappears, and bacillus ceases to be found in the sputum. The patients perceive a feeling of well-being after the injections, and state that they feel stronger and possess an amount of energy to which they had long been unaccustomed.

Maragliano's statistics[10] relate to 445 cases, including 82 that he reported in August, 1895, those recorded or reported by other Italian physicians, and a few contributed from France and Austria. The results summarised in the *Medical Journal* show the fever disappeared in 176 out of 332 cases, in 55 per cent. of cases of broncho-pneumonia with "microbian associations," in 32 per cent. of those of cavities, in 48 per cent. of those of diffuse broncho-pneumonia, and in 86 per cent. of those of circumscribed broncho-pneumonia. The local signs disappeared in 27 per cent., were improved in 41 per cent., were unchanged in 30 per cent., and were aggravated in 6 per cent. The writer in the *N.Y. Medical Journal* points out that these added together make 104; evidently there is a slip of the pen. There was an increase of weight in 57 per cent. The tubercle bacilli disappeared in 43·2 per cent. As a general rule one c c. of the serum was administered subcutaneously every other day. A case with very successful result is recorded by Jarrige,[11] and Raimondi and Mascucci[12] report the results of the Maragliano serum in five men and ten women. Five cases are described as phthisis with the lesion circumscribed in one or both lungs, slowly progressing disease with little or no fever, ten had more or less extensive terberculous broncho-pnemonia, with cavities, fever, and night sweats. No absolute cure was effected, but marked and persistent benefit was noted in four cases, transitory benefit in six cases. and no useful result in three cases. The total quantity of serum given in each case varied from 20 c.m. to 100 c.m.

[5] Presse Méd., June 10, 1896; Internat. Med. Mag., Dec., 1896; Central. für. Jour. Med., Oct. 24, 1896. [6] Deut. Med. Woch., April 1, 1897. [7] Med. Rec., April 11, 1896. [8] Rif. Med., Jan. 11, 1896; Epit. B. M. J., Apr., 18, 1896. [9] Prog. Med., Feb. 8, 1896; Epit. B. M. J., Apr. 18, 1896. [10] New York Med. Journ., edit., Aug. 15, 1896, cited from Zaeslein; Deut. Med. Zeit., July 27, 1896. [11] Compt. Rend de la Soc. de Biol., July 5, 1896. [12] Rif. Med. May 5, 1897; Epit. B. M. J., June 5, 1897.

